# Embedded Adaptive Admittance Control for a Modular Hip Exoskeleton Using Deep Learning-Based Gait-Phase Estimation

**DOI:** 10.3390/s26165008

**Published:** 2026-08-07

**Authors:** Tuan Anh Nguyen, Cong Phat Vo, Yekwang Kim, Youngbo Shim, Seung-Jong Kim

**Affiliations:** 1 Department of Biomedical Engineering, Korea University College of Medicine, Seoul 02841, Republic of Korea; gnob10@korea.ac.kr (T.A.N.); yekwang@korea.ac.kr (Y.K.); 2 T-Robotics Co., Ltd., Osan-si 18102, Republic of Korea; vcp3567@t-robotics.co.kr (C.P.V.); ybshim@t-robotics.co.kr (Y.S.)

**Keywords:** hip assistance robot, modular wearable robot, hip exoskeleton, adaptive admittance control, gait-phase awareness, deep learning

## Abstract

Hip assistance robots have become a practical solution as daily-life walking support. However, providing reliable and timely assistance during overground walking remains challenging because gait varies across users and walking conditions. This study presents HealBot-H, a newly developed 2-kg modular hip exoskeleton with a gait-phase-aware admittance control framework driven by a deep learning-based gait phase estimator. The robot features two active hip joints in the sagittal plane and uses a magnetic quick-release modular structure, enabling either unilateral or bilateral configuration depending on the application. The control framework combines a classical admittance law with a bidirectional long short-term memory network that estimates locomotion mode and the continuous gait phase from lower-limb inertial measurement units. The trained model was deployed on a Raspberry Pi 5 for real-time operation. Based on the estimated gait phase, the admittance parameters are scheduled across seven subphases of the gait cycle. To avoid abrupt torque changes at the subphase boundaries, a two-stage update rule comprising linear interpolation and exponential smoothing is applied. The robot was validated with ten healthy adults during overground walking. Surface electromyography (EMG) was recorded from five lower-limb muscles and compared between walking with and without the robot. The mean EMG reductions ranged from 19.0–23.9% over the gait cycle after false discovery rate correction (all $p_{\mathrm{FDR}}<0.01$). These results demonstrate that gait-phase-aware admittance control on a lightweight wearable platform can provide effective and well-timed walking assistance, while establishing a foundation for future personalized assistance.

## 1. Introduction

Driven by aging populations and the growing demand for mobility support, robotic exoskeletons have attracted considerable attention, with studies demonstrating their capacity to improve mobility and support independent living [1,2,3,4]. Among lower-limb assistive devices, hip assistance robots have emerged as a practical solution for daily use [5]. Because they require fewer actuators and sensors than multi-joint exoskeletons, these devices offer enhanced wearability for daily and long-term use. Recently, commercial hip assistance robots have exhibited a clear trend toward lightweight and wearable designs. Examples include GEMS-H [6] from Samsung Electronics Co., Ltd. (Suwon, Republic of Korea), WIM [7] from Wirobotics Co., Ltd. (Yongin, Republic of Korea), Angel Suit H10 [8] from Angel Robotics Co., Ltd. (Seoul, Republic of Korea), and HypershellX [9] from Hypershell Co., Ltd. (Shanghai, China). These systems typically weigh 1.6–3.0 kg and can be worn without assistance during daily walking. Consequently, recent developments in lightweight actuation, wearable structures, and portable power systems have shifted research efforts from hardware miniaturization toward the development of more adaptive and user-aware control strategies.

Human gait varies not only between individuals but also with walking speed and conditions. This becomes more complicated in real-world environments involving obstacles, ramps, or stairs. These variations render it difficult for a robot controller to provide consistent and appropriate assistance under all conditions. Previous studies have also reported that the timing [10,11,12] and magnitude [13,14] of the assistive force are important for the effectiveness of assistance. Therefore, the control strategy must be able to adjust assistance in real time when the user or the condition changes. For human–robot interaction (HRI), classical impedance and admittance control schemes are the most common [15,16,17]. In [18], an impedance method was used for assist-as-needed rehabilitation with a Takagi–Sugeno fuzzy observer to estimate the model uncertainties and disturbances. Impedance control regulates the force output in response to motion and works well under controlled conditions; however, it usually requires accurate modeling and careful tuning. By contrast, admittance control uses the interaction force as the input and produces the desired motion by adjusting the virtual inertia, damping, and stiffness. This compliant behavior makes admittance control well-suited for wearable applications targeting healthy or elderly users during daily walking. For example, Nagarajan et al. [17] presented an admittance-based hip controller that changes the dynamic response of a human–robot system without assuming strict gait periodicity. Accordingly, admittance control is adopted as the foundation of the proposed framework. However, a single fixed admittance parameter set forces a compromise between compliance and stability. Low virtual inertia and damping increase responsiveness but reduce stability, whereas high values improve stability at the cost of larger interaction forces [19,20,21]. This limitation is more pronounced during walking, because the biological hip moment changes continuously within the gait cycle, and the effectiveness of assistance depends strongly on its timing across the cycle [10,14,22]. Therefore, a phase-dependent adaptive scheme is adopted in this study.

In addition to force generation, accurate gait phase information is important for synchronizing assistance with user motion. Classical methods such as finite-state machines [23,24], time-based estimation [25,26], phase oscillators [27], and adaptive oscillators [28,29] are widely used owing to their simplicity and low computation costs. However, these methods can be affected by sensor noise, gait variability, and changes in the walking conditions. Therefore, recent studies have shifted to machine learning and deep learning approaches for gait phase estimation [11,30], locomotion mode classification [31], and walking condition identification to enable personalized assistance [32,33,34,35]. Qian et al. [31] and Tricomi et al. [36] used vision-based methods for real-time locomotion mode classification during exoskeleton-assisted walking. Kang et al. [37] used a neural network with inertial measurement unit (IMU) and hip encoder signals to estimate the gait phase at different walking speeds. Although these approaches can improve timing and awareness, many rely on computationally intensive models or external equipment, which can hinder deployment on lightweight wearable platforms. Deep learning-based gait phase estimation is well established at the algorithmic level; however, its real-time implementation in lightweight wearable hip robots, particularly when integrated with phase-resolved adaptive control, remains scarce in the literature. This gap is addressed in the present study.

This study presents HealBot-H, a 2-kg modular hip assistance robot with a gait-phase-aware admittance control framework driven by a deep learning-based gait phase estimator. HealBot-H is a new platform that was designed and fabricated for this study. In the proposed framework, the virtual inertia, damping, and stiffness of the admittance law were scheduled across seven biomechanical subphases of the gait cycle, and a two-stage update rule combining linear interpolation with exponential smoothing was applied to ensure continuous parameter updates at the subphase boundaries. The robot was designed in a modular form with a magnetic quick-release structure, allowing a unilateral or bilateral configuration depending on the application. The bidirectional long short-term memory (Bi-LSTM) model used in the gait phase estimator was developed and validated offline in our previous work [38], whereas the present study focuses on its embedded real-time deployment on Raspberry Pi 5 (RPi 5) (Raspberry Pi Ltd., Cambridge, UK) using Open Neural Network Exchange (ONNX) Runtime (v1.21.0). Real-time admittance control and safety monitoring were implemented in C++14, and the gait phase estimator achieved an average inference time of approximately 2 ms per cycle. The integrated system was validated in ten healthy adults during overground walking without subject-specific tuning. The scope of this study is the design of the platform, the real-time implementation of the controller, and its validation during level overground walking with healthy adults. This design supports daily walking assistance while providing a foundation for asymmetric gait conditions and future personalized control.

The remainder of this study is organized as follows: Section 2 describes the hardware design of HealBot-H and the proposed gait-phase-aware admittance control framework. Section 3 presents the results of both the controller behavior trial and EMG validation. Section 4 discusses and outlines the directions for future research. Finally, Section 5 concludes the study.

## 2. Materials and Methods

### 2.1. Design of HealBot-H

HealBot-H is designed as a lightweight modular hip assistance robot to support walking in daily life. The mechanical design aimed to keep the total weight low for long-term wearability, while providing sufficient joint torque to assist healthy users during overground walking. The final prototype weighs 2-kg including a battery.

**Figure 1:**
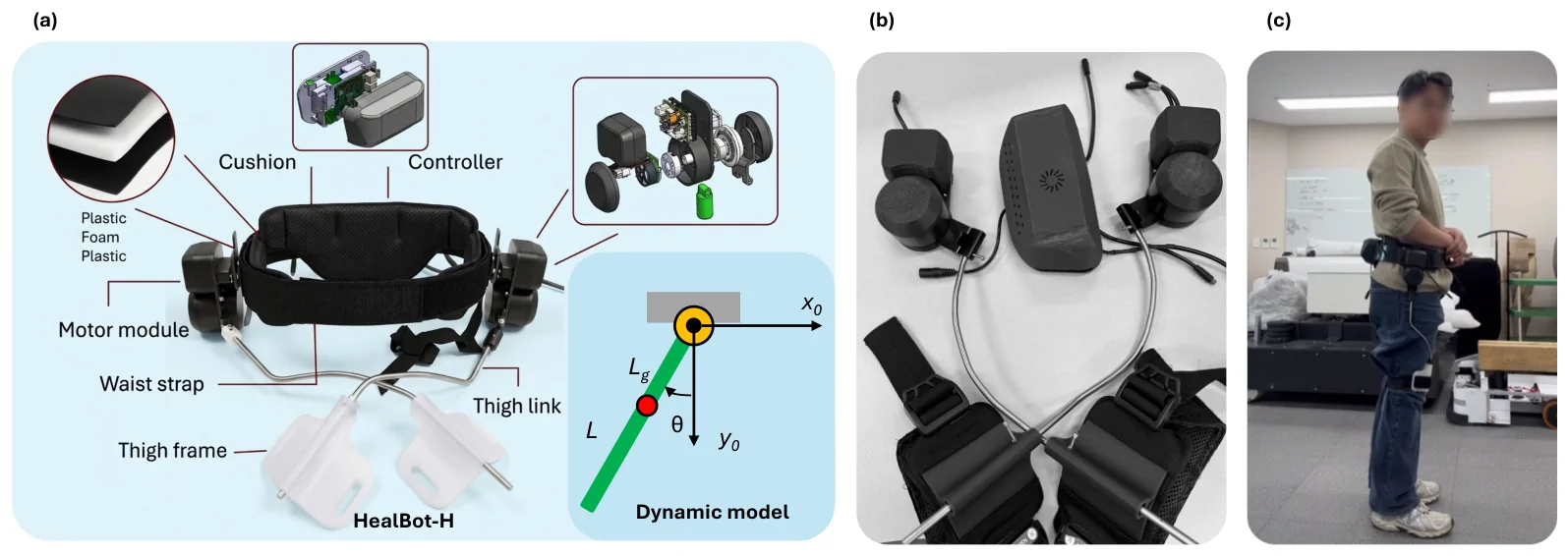
Hardware overview of HealBot-H. (**a**) Main components of the robot, including the waist frame, two actuator modules, control unit, and thigh attachment units. (**b**) Modular configuration with actuator and robot controller modules. (**c**) User wearing the robot in bilateral configuration.

As shown in Figure 1a, the robot consists of a flexible waist frame, two actuator modules, one control unit, and two thigh-attachment units. Each actuator module was mounted on one side of the waist frame, aligned approximately with the user’s hip joint axis, and connected to a stainless-steel thigh link that transmitted the assistive torque to the user’s leg. Each hip joint provided one active degree of freedom (DOF) for flexion and extension in the sagittal plane driven by the actuator. Additionally, one passive DOF allows abduction and adduction so that the natural pelvic motion during walking is not constrained. Modularity is enabled by magnetic quick-release connectors that combine a magnetic holding force with a mechanical locking pin, and users can assemble or disassemble the robot in less than 10 s, as measured during pilot donning tests. The same waist frame can be configured for unilateral or bilateral use, and different actuator modules with different torque ranges can be attached to the same frame, depending on the application. Figure 1b shows the modular configuration of HealBot-H and Figure 1c shows a user wearing the robot in a bilateral configuration.

Each actuator module uses a brushless DC motor (Maxon ECXFL32L, Maxon, Sachseln, Switzerland, 24 V, 90 W) combined with a harmonic drive gearbox with a reduction ratio of 1:100, which provides a continuous joint torque of approximately 10.3 Nm and a peak joint torque of approximately 43.8 Nm after gear reduction. According to [4,15], this torque range is comparable with reported lightweight hip robots designed for daily walking. The joint angle was measured using an incremental encoder integrated into each motor. Five IMUs (WT9011DCL-RF, WitMotion Shenzhen Ltd., Shenzhen, China) provided signals for the gait phase estimator described in Section 2.2. No force or torque sensors are used in the system. The human–robot interaction torque was estimated by a model-based observer from the measured motor current and joint kinematics, as described in Section 2.3. This sensorless approach reduces system weight, hardware complexity, and integration requirements.

The control system was centered on RPi 5, which served as the central processing unit for HealBot-H. Real-time admittance control and safety monitoring were implemented in C++ and executed in a 100 Hz control loop, while gait phase estimation was performed by an ONNX Runtime inference module running on the same processor. The RPi 5 communicates with two Maxon EPOS4 Compact 50/5 Controller Area Network (CAN) motor drivers (Maxon, Sachseln, Switzerland) via a CAN interface, exchanging current commands and receiving motor position and motor current feedback for closed-loop control. The overall hardware specifications of HealBot-H are listed in Table 1.

### 2.2. Gait Phase Estimation

The proposed controller uses gait phase information to schedule assistance behaviors in real-time. HealBot-H supports two gait-phase estimation methods. The first is a conventional rule-based estimator that uses joint encoder signals and finite-state machine logic to identify the current subphase from local joint kinematics. The second is a deep learning estimator that uses five lower-limb IMU signals and a Bi-LSTM network. Both methods produced the same output structure, namely the current locomotion mode and continuous gait phase progression, as a percentage of the cycle. During steady cyclic walking, both estimators track the gait phase reliably, and the rule-based estimator is therefore retained as a safety fallback when the inertial data stream is unavailable. The deep learning estimator is used as the primary phase source because it extends beyond this condition. It identifies the transition between walking and standing through the locomotion mode classifier, and it covers multiple locomotion modes from the same inertial signals without per-terrain threshold tuning.

The deep learning estimator receives signals from five IMUs attached to both thighs, both shanks, and the lower abdomen. The thigh and shank units measure the motion on either side of the knee, which covers the stance and swing transitions. The lower-abdomen unit provides a pelvis-reference signal for bilateral timing. Each IMU provided six channels: three-axis linear acceleration and three-axis angular velocity. The magnetometer of each unit was not used because the model estimates the gait phase from the six-axis inertial pattern rather than from an absolute orientation. The IMU signals were stored in a rolling buffer and processed using a 500 ms (50 frames) sliding window with a 10 ms (1 frame) stride. The windowed data were then fed into a two-stage deep learning model. Because the model operates on these raw acceleration and angular velocity signals rather than on reconstructed segment orientations, small placement or alignment variations between donning sessions have little effect on the phase output. Both stages were trained with the Adam optimizer and early stopping, and the hyperparameters were selected by random search. The Bi-LSTM model used in this study was developed in our previous study [38]. The model was validated with a leave-one-subject-out procedure over ten healthy participants, achieving a median phase classification accuracy of 94.0% and a phase progression root mean square error of 7.6%. This value is the error of the phase progression within each phase rather than the error over the whole gait cycle, for which root mean square errors of approximately 4% to 5% are reported for real-time estimators [32]. The two-stage update rule described in Section 2.4 maintains a continuous parameter transition under this level of estimation error. This model was deployed in real time on the embedded controller of HealBot-H using ONNX Runtime, with an average inference time of approximately 2 ms per cycle. This is well below the 10 ms control period of the 100 Hz loop, leaving sufficient computational headroom for the remainder of the control pipeline. Although the model supports four locomotion modes at the algorithmic level, this study focuses exclusively on standing and level walking, leaving the remaining modes for multiterrain validation in future work.

**Figure 2:**
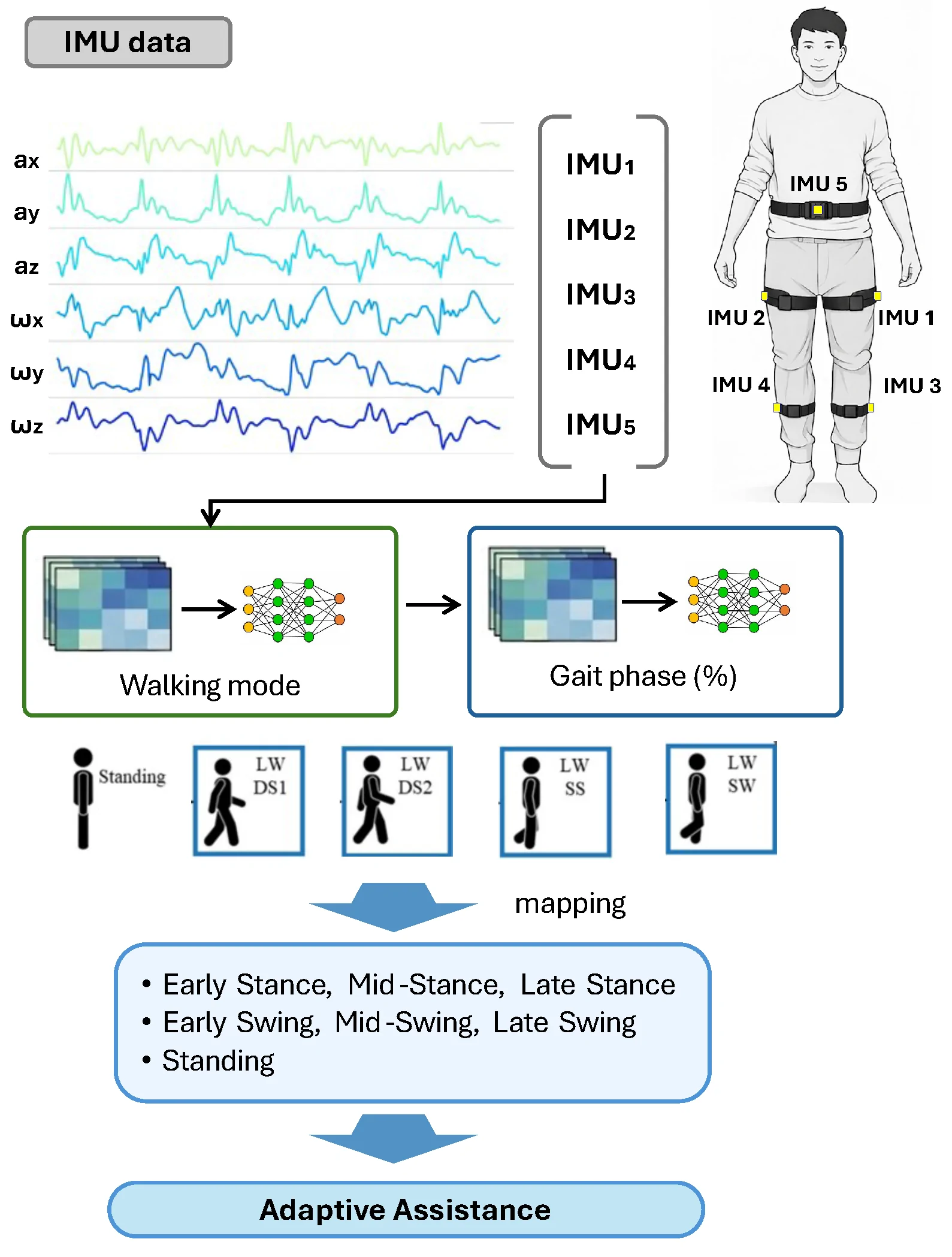
Gait phase estimation pipeline. Six-axis signals (three-axis acceleration $a_{x}$, $a_{y}$, $a_{z}$ and three-axis angular velocity $\omega_{x}$, $\omega_{y}$, $\omega_{z}$) from five IMUs (IMU_1_–IMU_5_) attached at the thighs, shanks, and lower abdomen are processed by a two-stage Bi-LSTM model. The first stage classifies the locomotion mode, and the second stage regresses the continuous gait phase (%). The mode and phase output is then mapped to the seven discrete subphases used by the adaptive assistance controller.

The overall pipeline of the gait-phase estimation framework is shown in Figure 2. Within the controller, the continuous phase progression output is mapped to seven discrete subphases of the gait cycle following standard biomechanical conventions [39]. These subphases were defined as Early Stance, Mid-Stance, Late Stance, Early Swing, Mid-Swing, Late Swing, and Standing. This seven-subphase representation captures phase-dependent variations in hip-joint biomechanics throughout the gait cycle, enabling more granular adaptation of the admittance parameters than a binary stance–swing label. This serves as the input to the gain scheduler described in Section 2.4, where the biomechanical rationale for each subphase is detailed.

### 2.3. Adaptive Admittance Control

**Figure 3:**
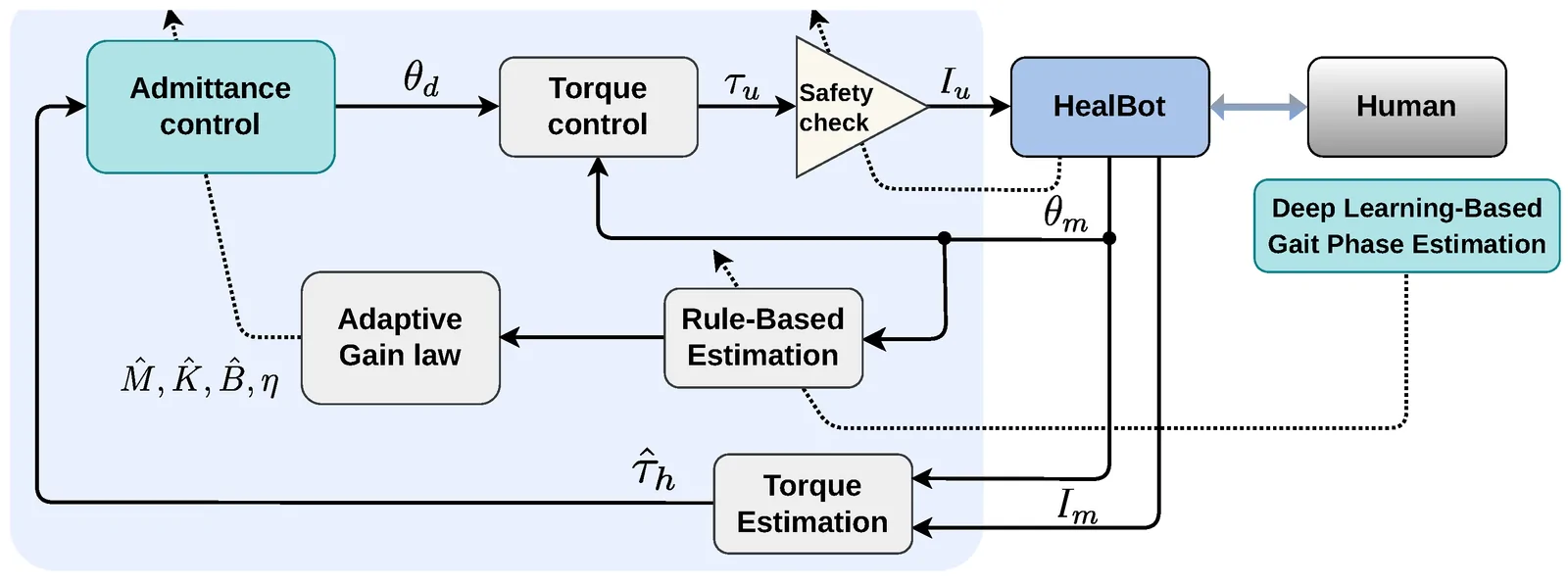
Block diagram of the closed-loop control scheme.

The proposed control architecture is shown in Figure 3. The system operates in a closed loop and comprises six functional modules: interaction torque estimation, gait phase estimation, adaptive gain schedulhip-assistance roboting, admittance control, low-level torque control, and safety monitoring. The admittance controller generates the desired joint trajectory $\theta_{d}$, which is tracked by the low-level torque controller to produce a command torque $\tau_{u}$. This command passes through a safety check module before being dispatched as a motor current command $I_{u}$ to the HealBot-H actuator. The resulting joint motion $\theta_{m}$ and motor current $I_{m}$ are fed back to the torque estimation and gait phase estimation modules to close the loop.

A key feature of this architecture is that the adaptive gain-scheduling module concurrently receives two real-time inputs from the closed loop: the estimated interaction torque and estimated gait phase. Combining these two signals allows the controller to adapt assistance based on both the user’s interaction force and the current phase within the gait cycle. The combined adaptation mechanism constitutes the core strategy of the proposed control framework.

No dedicated force or torque sensors were used to reduce the system weight and hardware complexity. Instead, the HRI torque is estimated via a model-based dynamic observer using the measured motor current and joint kinematics. The estimated interaction torque ${\hat{\tau }}_{h}$ is derived from the joint dynamic equation [40].

(1) $${\hat{\tau }}_{h}=I{\ddot{\theta }}_{m}+B{\dot{\theta }}_{m}+\tau_{g}\left(\theta_{m}\right)+\tau_{f}\left({\dot{\theta }}_{m}\right)-\tau_{m}$$

where $\theta_{m}$ is the measured joint angle, $\tau_{m}$ is the motor-generated torque. Terms *I* and *B* denote the equivalent joint inertia and viscous damping coefficient, respectively. The gravity torque is modeled as $\tau_{g}=mgL_{g}\cos\left(\theta_{m}\right)$ and Coulomb friction is approximated as $\tau_{f}=c\;\mathrm{sign}\left({\dot{\theta }}_{m}\right)$, where *c* is the friction coefficient [41]. The parameters *I*, *B*, *m*, $L_{g}$, and *c* were identified from the robot experiments.

The core assistance strategy was based on admittance control, which maps the estimated interaction torque to the desired joint trajectory using a virtual mechanical system. The admittance control law is formulated as,

(2) $$\eta {\hat{\tau }}_{h}=\hat{M}\left(\theta \right){\ddot{\theta }}_{d}+\hat{B}\left(\dot{\theta },\theta \right){\dot{\theta }}_{d}+\hat{K}\left(\theta_{d}-\theta_{0}\right)$$

where $\theta_{d}$ is the desired joint angle, $\theta_{0}$ is the neutral joint position, and $\hat{M}$, $\hat{B}$, $\hat{K}$ represent the virtual inertia, damping, and stiffness parameters, respectively. The scalar η is the assistance gain, which modulates the weighting of the estimated user torque in generating the assistive motion. The desired joint trajectory $\theta_{d}$ is tracked by a low-level controller that computes the motor torque command.

### 2.4. Adaptive Gain Scheduling

The admittance control performance depends critically on the selection of the virtual parameters $\hat{M}$, $\hat{B}$, and $\hat{K}$, which determine the dynamic characteristics of the HRI. Prior work has shown that these parameters involve an inherent trade-off between compliance and stability, where lower virtual inertia and stiffness increase responsiveness to user intent but may reduce stability, whereas higher values improve robustness at the expense of perceived resistance [21,42]. The mechanical demands of the hip joint vary substantially within the gait cycle. The early stance corresponds to load acceptance, the late stance corresponds to push-off, and the swing phase is further divided into limb acceleration, mid-swing transit, and limb deceleration. However, a single fixed parameter set cannot satisfy these requirements. Based on this observation, the proposed strategy assigned an independent set of admittance parameters to each of the seven subphases of the gait cycle. The parameter values were selected through a trial-and-error procedure in pilot experiments with healthy participants during level walking with HealBot-H, guided by established biomechanical principles and admittance control design considerations reported in [43,44]. Accordingly, the virtual inertia and stiffness were minimized during the swing phase to facilitate limb advancement, whereas the virtual damping and stiffness were increased during the stance phase to support load transfer and postural stability.

The proposed gain scheduler prevents discrete switching between fixed parameter sets to avoid abrupt torque fluctuations during the transition between subphases. Instead, the parameters are updated continuously in each control cycle using a two-stage rule. In the first stage, when the estimated gait phase progression exceeds 80% within the current subphase, linear interpolation generates a target parameter vector ${\hat{\Theta }}_{t}$ that combines the current subphase set with the next subphase set:

(3) $${\hat{\Theta }}_{t}=\left(1-\beta \right)\;{\hat{\Theta }}_{i}+\beta \;{\hat{\Theta }}_{i+1}$$

where $\hat{\Theta }={\left[\hat{M},\hat{B},\hat{K},\eta \right]}^{⊤}$ denotes the parameter vector, *i* is the current subphase, $\beta =\max\;\left(0,\;\frac{p-80}{20}\right)\in \left[0,1\right]$ is the interpolation weight, with *p* as the gait phase percentage within the current subphase. When p≥80% the target ramps linearly toward the next subphase value ${\hat{\Theta }}_{i+1}$, providing an anticipatory transition before the subphase boundary is reached. In the second stage, the actual parameter vector $\hat{\Theta }\left(t\right)$ is advanced one step toward the target through first-order exponential smoothing:

(4) $$\hat{\Theta }\left(t\right)=\hat{\Theta }\left(t-dt\right)+\alpha \;\left({\hat{\Theta }}_{t}-\hat{\Theta }\left(t-dt\right)\right)$$

where dt is the control time step and $\alpha =1-e^{-\lambda \;dt}\in \left[0,1\right]$ is the smoothing coefficient governed by the transition rate λ. This stage removes the remaining discontinuities in the target trajectory and prevents discontinuities in the applied parameter vector. Therefore, the admittance parameters are not fixed during operation but evolve continuously in response to the real-time gait phase estimate, producing an assistance profile that adapts smoothly across the gait cycle. Without these two-stages, the controller switches discretely between parameter sets, and a step change in the virtual inertia, damping, and stiffness propagates directly into the assistive torque at every subphase boundary.

### 2.5. Experiments

#### 2.5.1. Experimental Protocols

Ten healthy adults were recruited for this study (6 males and 4 females; age: 32.5±4.6 years; height: 165.4±6.3 cm; weight: 66.8±16.2 kg; values given as mean ± standard deviation). All participants were capable of independent ambulation without assistive devices and presented with no neuromuscular, orthopedic, or cardiovascular conditions that could affect their gait. Each participant was fully informed of the purpose of the study, procedures, and potential risks and subsequently provided written informed consent. The experimental protocol was approved by the Institutional Review Board (IRB) of the Korea University College of Medicine (IRB No. KUIRB-2025-0315-01) and conducted in accordance with the Declaration of Helsinki.

Surface EMG signals were recorded from five lower-limb muscles on the right leg: the rectus femoris (RF), biceps femoris (BF), vastus medialis (VM), soleus (SL), and tibialis anterior (TA). These muscles were selected because they cover the main functional groups of the lower limbs while walking. The EMG signals were recorded using a Delsys Trigno wireless system (Delsys Inc., Boston, MA, USA) at a sampling rate of 1777 Hz for the EMG channels and 74 Hz for the inertial signals integrated into each Trigno sensor.

The experiment was performed on a flat overground walkway with a total distance of approximately 40 m. Each participant performed four passes of 10 m straight walking at a self-selected comfortable speed. Overground walking was deliberately selected, as it replicates daily gait patterns more closely than treadmill walking. Two conditions were tested in a fixed order: the *No-HealBot-H* condition followed by the *HealBot-H* condition, so that the baseline natural gait was recorded before any familiarization with the robot. In the *No-HealBot-H* condition, the participants walked without the robot as a baseline. In the *HealBot-H* condition, the participant walked while wearing the robot with the proposed controller active, and the assistive torque was capped at ±2 Nm for safety and comfort. Sufficient rest periods were provided between the conditions to prevent physical fatigue, and a short familiarization session preceded the HealBot-H trials, as mandated by the approved IRB protocol.

In addition to the EMG validation experiment, a separate walk–turn–walk trial was conducted to demonstrate the controller behavior across continuous walking and standing transitions. The trial consisted of two continuous walking bouts on level ground separated by a brief standing interval for a total duration of 30 s. The hip joint angles, assistive torque, joint angular velocity, and estimated gait phase were logged at a control loop rate. The results of this demonstration are presented in Section 3.

#### 2.5.2. Data Processing

The EMG signals were segmented into individual gait cycles using the mediolateral angular velocity obtained from the right-shank IMU. Foot-contact and foot-off events were detected from the zero crossings around each mid-swing local minimum following the procedure in [45]. Each EMG signal was divided into the stance phase (from foot contact to foot off) and the swing phase (from foot off to foot contact). The raw EMG signals were bandpass-filtered using a fourth-order Butterworth filter with a passband of 20–500 Hz. To obtain an envelope for muscle activation, the filtered signal was converted to its waveform-length (WL) representation [46,47], which is a well-established feature for real-time EMG analysis [48,49]. EMG WL was used as an indicator of muscle activation in all subsequent analyses. The WL values were compared directly between the *No-HealBot-H* and *HealBot-H* conditions for the same participant.

To assess whether HealBot-H assistance reduced the muscle demand, the *No-HealBot-H* and *HealBot-H* conditions were compared at two levels. The individual-level analysis compared the mean EMG WL of each gait cycle between the two conditions on a per-subject basis using Student’s *t*-test. Group-level analysis tested subject-level percentage change values against zero using the Wilcoxon signed-rank test. The percentage of change in each muscle was calculated as follows:

(5) $$\Delta \mathrm{EMG}\;{\mathrm{WL}}_{\mathrm{rel}}=\frac{{\mathrm{mean}}_{\mathrm{HealBot}-H}-{\mathrm{mean}}_{\mathrm{No}-\mathrm{HealBot}-H}}{{\mathrm{mean}}_{\mathrm{No}-\mathrm{HealBot}-H}}\times 100\%$$

The non-parametric test was used because the sample size was small and the percent-change distributions did not consistently satisfy the normality assumption. To control for false positives across 15 multiple comparisons (5 muscles × 3 windows), Benjamini–Hochberg false discovery rate (FDR) correction was applied [50]. Adjusted *p*-values were denoted $p_{\mathrm{FDR}}$, and significance levels were reported as $p_{\mathrm{FDR}}<0.05$, $p_{\mathrm{FDR}}<0.01$, and $p_{\mathrm{FDR}}<0.001$. All signal processing and statistical analysis were performed using custom scripts written in MATLAB R2025a (MathWorks Inc., Natick, MA, USA).

## 3. Results

### 3.1. Controller Behavior During Walking

**Figure 4:**
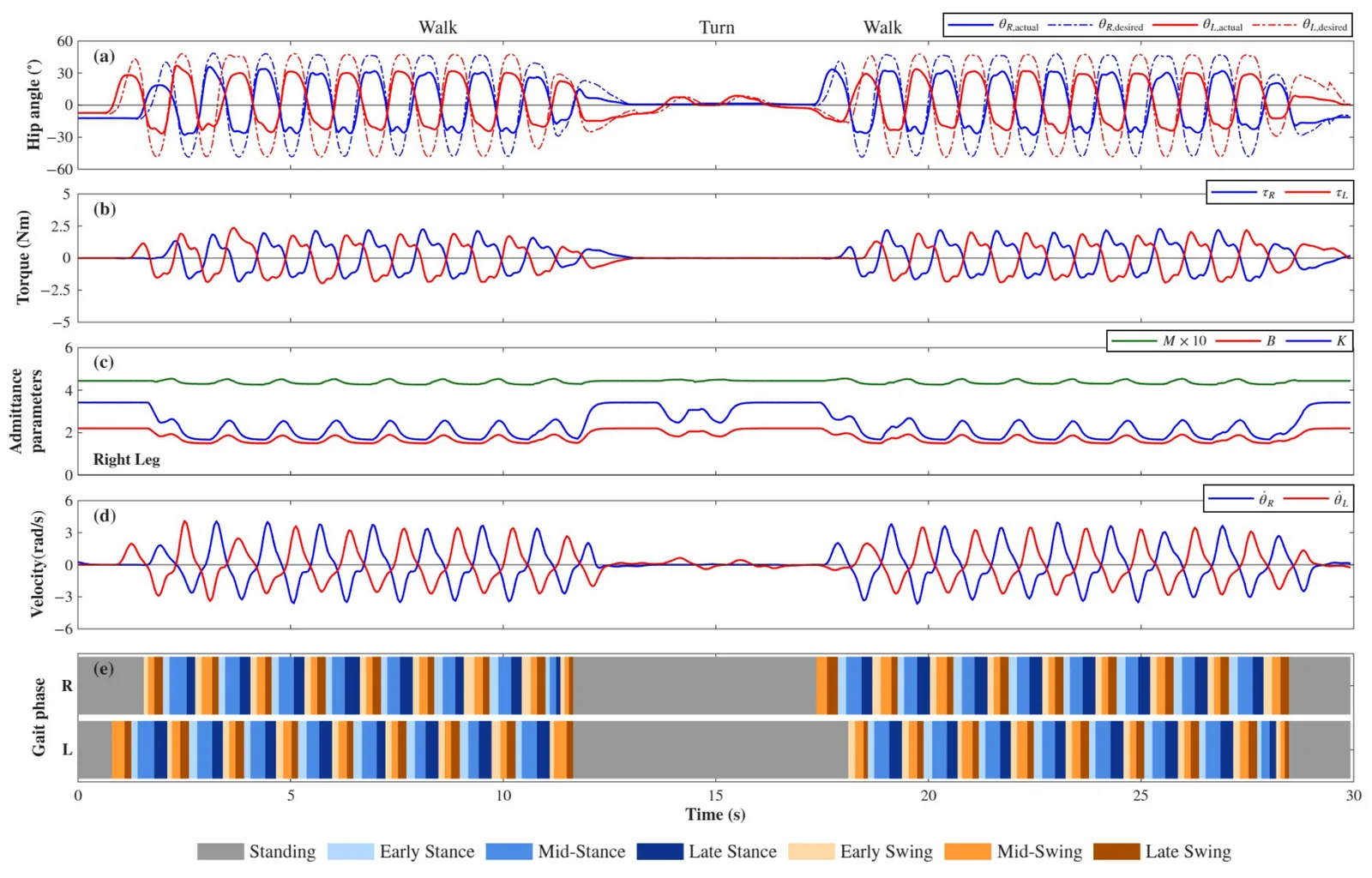
Walking performance of the proposed adaptive admittance controller during a representative walk–turn–walk trial. The 30 s window contains two continuous walking bouts separated by a brief standing interval. (**a**) Bilateral hip joint angles: dashed lines show the desired joint trajectories generated by the admittance dynamics ($\theta_{d}$), and solid lines show the measured joint angles ($\theta_{\mathrm{actual}}$). (**b**) Motor assistive torque. (**c**) The admittance parameters for the right leg. (**d**) Hip joint angular velocity. (**e**) Gait phase estimated by the gait phase estimator, color-coded for the seven subphases (Standing, Early/Mid-/Late Stance, Early/Mid-/Late Swing).

Figure 4 shows the hip kinematics, motor-assistive torque, admittance parameter evolution, and gait phase identification recorded during the overground walk–turn–walk trial. The trial consisted of two continuous walking bouts separated by turnaround interval. Figure 4a shows the bilateral hip joint angles. The desired trajectory $\theta_{d}$ had a wider envelope than the measured angle $\theta_{\mathrm{actual}}$, which is the expected behavior of an admittance controller. $\theta_{d}$ represents the motion that the virtual mechanical system would produce without the user’s biomechanical resistance, while $\theta_{\mathrm{actual}}$ reflects the closed-loop interaction with the user’s own gait dynamics. The controller did not track $\theta_{d}$ as a kinematic reference. Instead, $\theta_{d}$ is an internal state variable used to compute the assistive command. The assistive torque shown in Figure 4b was delivered only during walking bouts and attenuated to near zero whenever the system transitioned into the standing state, which confirmed that the adaptive gain scheduler effectively suppressed unnecessary assistance when the user was not walking.

Figure 4c shows the real-time evolution of the admittance parameters *M*, *B*, and *K* for the right leg during the same trial, where *M* was plotted at ten times its actual value for enhanced visual clarity. These three parameters were continuously modulated across the gait cycle in response to the estimated phase. During the turnaround interval, all three parameters settled at the values assigned to the standing subphase and smoothly returned to the walking modulation pattern upon the initiation of the second walking bout. The absence of abrupt transitions or step-like discontinuities at the subphase boundaries indicates that the proposed two-stage update rule achieves smooth parameter transitions, eliminating the risks of discrete switching. The hip joint angular velocity in Figure 4d exhibits smooth periodic profiles without high-frequency oscillations or step discontinuities, which is consistent with the absence of abrupt torque changes, as shown in Figure 4b. Finally, the deep learning gait phase classifier shown in Figure 4e correctly identified both distinct walking bouts and standing intervals without producing any spurious gait cycles. Throughout each gait cycle, the stance and swing subphases progressed according to the expected biomechanical sequence for both lower limbs. The right- and left-phase trajectories exhibited distinct out-of-phase relationships, consistent with healthy bilateral coordination.

**Figure 5:**
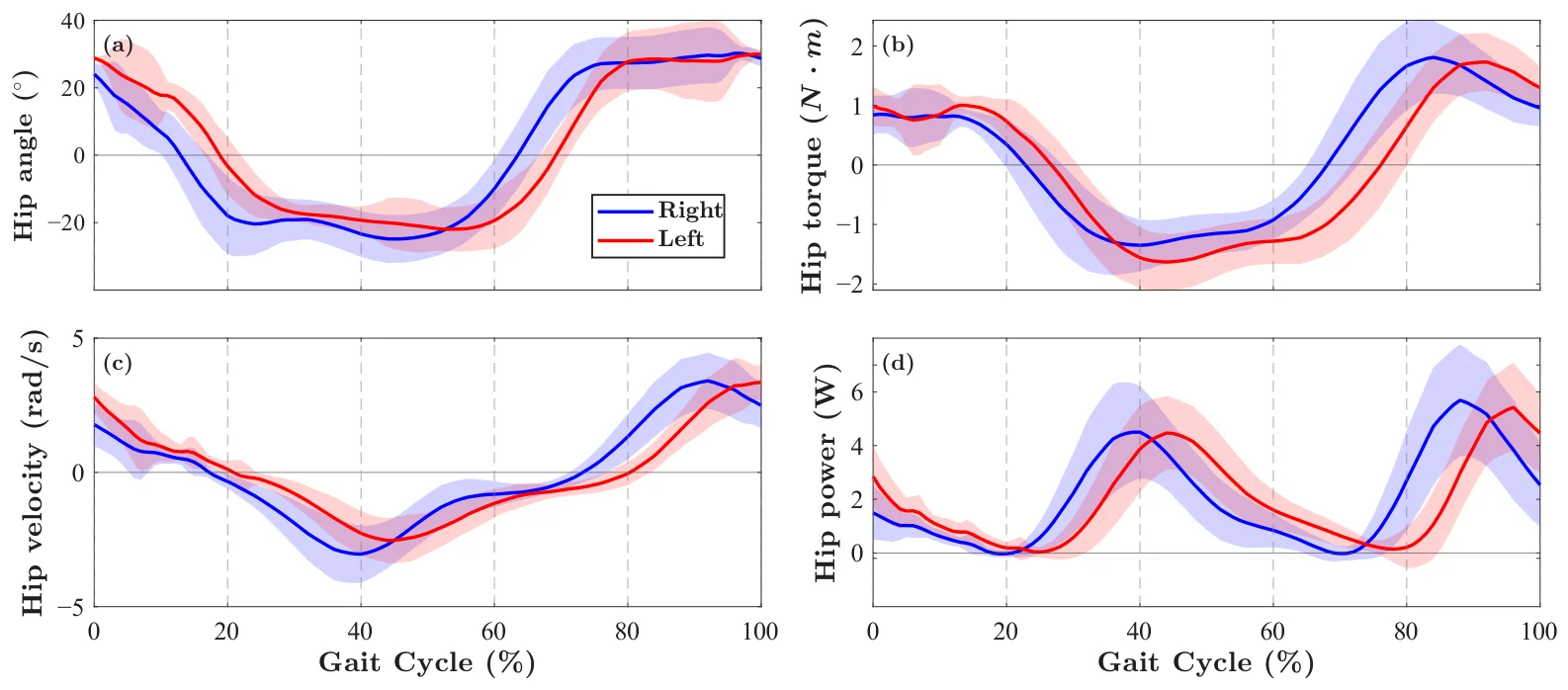
Ensemble-averaged hip joint profiles. Mean (solid line) and standard deviation (shaded band) of (**a**) hip joint angle, (**b**) motor assistive torque, (**c**) hip angular velocity, and (**d**) hip mechanical power. Blue and red traces denote the right and left legs, respectively.

The ensemble-averaged hip kinematics, assistive torque, joint angular velocity, and mechanical power across the normalized walking gait cycle are shown in Figure 5. The shaded standard deviation bands are narrow across all four signals, indicating that the controller output is repeatable from cycle to cycle.

The mechanical power profile in Figure 5d provides the clearest evidence that the assistance was well timed with the user’s motion. A positive mechanical power indicates that the motor torque acts in the same direction as the hip angular velocity, whereas a negative power indicates energy absorption. The recorded power was positive for almost the entire gait cycle, with only brief and small negative excursions during the early stance and early swing transitions. Two main positive-power regions are observed. The first peak occurred during late stance, at approximately 37.5±9.5% and 42.0±10.1% of the gait cycle for the right and left legs, respectively. The second peak occurred during late swing, at approximately 89.1±4.4% and 95.2±3.2% of the gait cycle. These two regions correspond to the late-stance push-off and late-swing limb-deceleration phases, the two periods during which the hip joint naturally generates positive mechanical power during healthy walking. The alignment between the assistive power peaks and these biomechanical events suggests that the controller produced well-timed assistance that was synchronized with the user’s natural walking dynamics.

The proposed controller was compared with a fixed-parameter admittance controller under the same protocol, in which the admittance parameters were held constant at the mid-stance values. The fixed-parameter controller identified the gait subphases correctly during steady walking. However, the interaction force between the user and the device increased across both walking bouts, which indicates that the device resisted the movement of the user more than the proposed controller did. The fixed-parameter controller also sustained assistive torque during the turnaround interval, where no cyclic gait pattern was present. A single parameter set therefore does not cover non-cyclic events. The full comparison is provided in Supplementary Materials, Figures S1 and S2.

### 3.2. Muscle Activation Reduction During Assisted Walking

**Figure 6:**
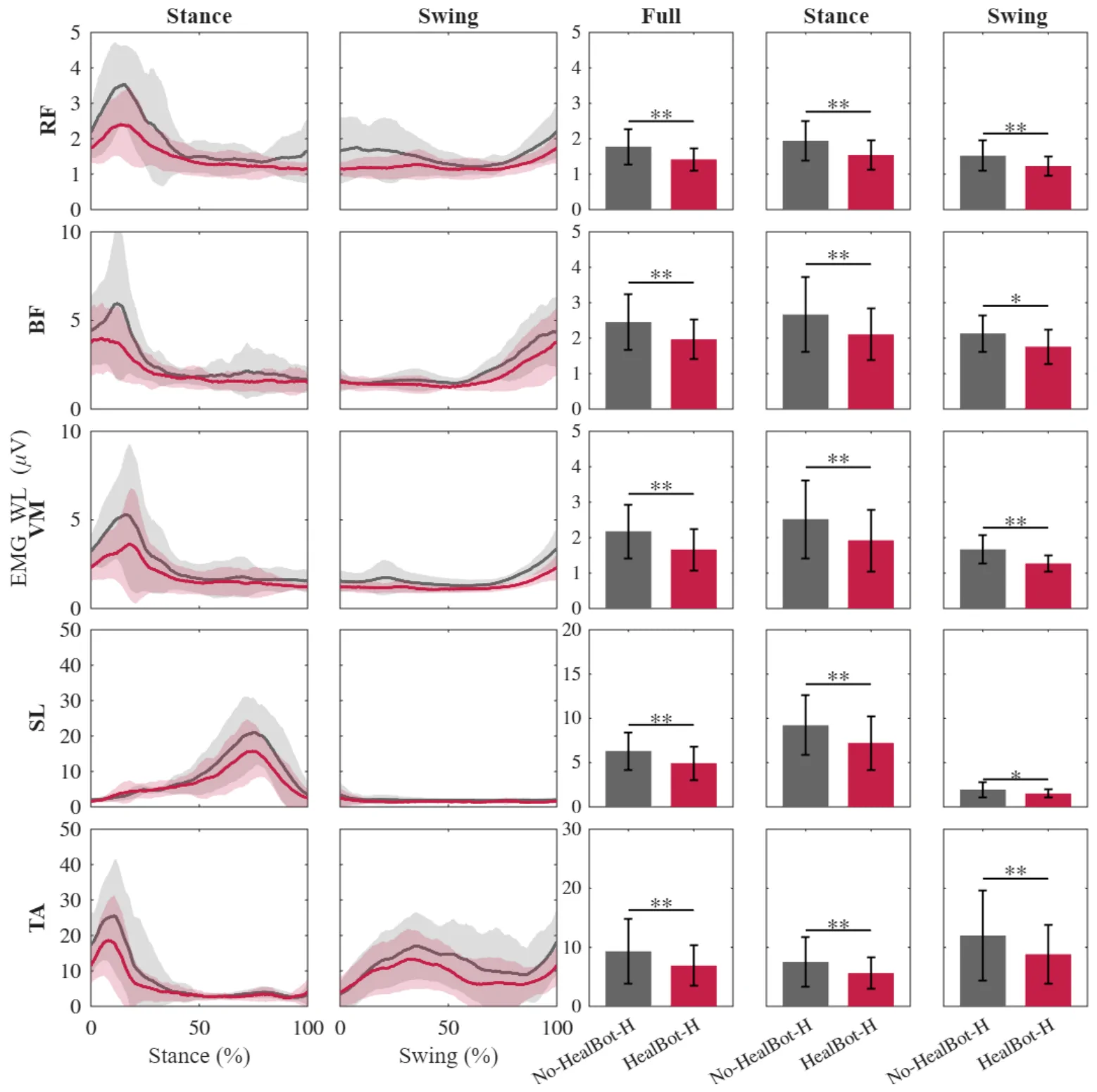
Group-level EMG comparison between the *No-HealBot-H* and *HealBot-H* conditions across five lower-limb muscles (RF, BF, VM, SL, TA). The first two columns show the mean ± standard deviation (SD) waveform of EMG WL across all participants during the stance and swing phases (n=10). The next three columns show bar plots of mean ± SD EMG WL amplitude during the full gait cycle, stance phase, and swing phase, respectively. Gray and red indicate the *No-HealBot-H* and *HealBot-H* conditions, respectively. Significance markers above each bar pair show the Wilcoxon signed-rank test result on subject-level percent change after Benjamini–Hochberg FDR correction (* $p_{FDR}<0.05$, ** $p_{FDR}<0.01$ ).

Surface EMG was compared between the *No-HealBot-H* and *HealBot-H* conditions for all five lower-limb muscles at three levels: full gait cycle, stance phase, and swing phase. Figure 6 presents group-level EMG comparisons, where the first two columns delineate the EMG WL profiles during stance and swing, and the remaining three columns show the mean amplitudes computed for the full cycle, stance, and swing. For every muscle and window, the EMG WL was lower in the *HealBot-H* condition, whereas the waveform shape was preserved, indicating that the controller reduced the activation magnitude without altering the activation timing.

A comparison of the mean EMG WL values during the stance phase provides the most biomechanically meaningful evaluation of the proposed controller because all five target muscles are functionally active during stance, contributing to body weight support, load transfer, and push-off. All five muscles showed statistically significant reductions in stance-phase EMG WL (all $p_{\mathrm{FDR}}<0.01$) with rank-biserial effect sizes between −0.85 and −0.89. The detailed values for each muscle are listed in Table 2. The same reduction pattern was observed over the full gait cycle, where the RF, BF, VM, SL, and TA decreased by 18.97%, 19.09%, 23.33%, 21.91%, and 23.88%, respectively. The consistency between the full cycle and stance-phase reductions indicated that most of the assistance effect on muscle activation was concentrated in the stance phase.

During the swing phase, the reductions were statistically significant for all five muscles ($p_{\mathrm{FDR}}<0.01$). SL is naturally inhibited during swing because the limb is unloaded and the ankle is dorsiflexing, so the baseline SL activity during swing is already very low (1.96±0.88 μV, compared with 9.22±3.39 μV during stance). A relative reduction in a very small baseline is less biomechanically meaningful than a reduction in a large baseline. By contrast, TA is naturally very active during swing for foot clearance and showed a clear reduction of 24.24±12.52% ($p_{\mathrm{FDR}}<0.01$), which reflects a meaningful decrease on a substantial baseline.

**Figure 7:**
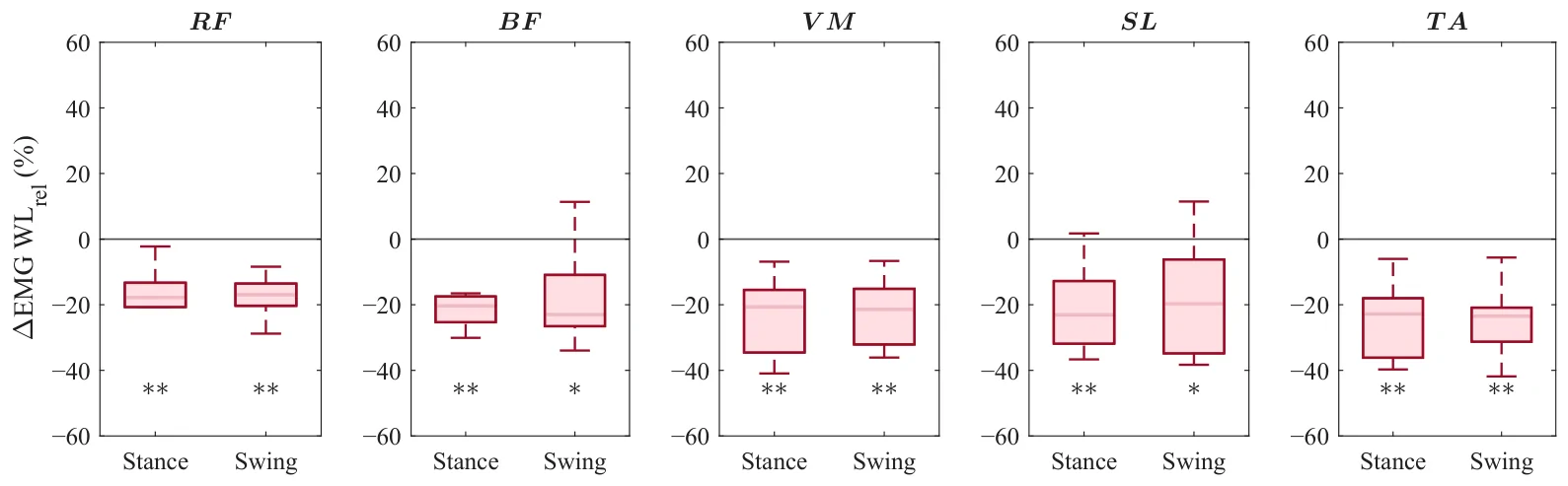
Group distribution of the percent change in EMG WL ($\Delta \mathrm{EMG}\;{\mathrm{WL}}_{\mathrm{rel}}$) per muscle and per phase across the 10 participants (n=10). Box plots show the percent change for RF, BF, VM, SL, and TA during the stance and swing phases. Data are shown as median (line within box), 25th and 75th percentiles (box edges), and minimum and maximum values (whiskers). Significance markers above each box pair show Wilcoxon signed-rank test results on subject-level percent change (* $p_{FDR}<0.05$, ** $p_{FDR}<0.01$).

The group distribution of the percentage change values across the ten participants is shown in Figure 7. For all five muscles and both phases, the median percentage change was clearly below zero, with most boxes lying between −19% and −24%. The whiskers also remained below zero in most cases, and no participant showed an EMG WL increase greater than a few percent. This consistent negative distribution across participants indicates that the observed reductions were consistently distributed throughout the cohort rather than being driven by a small number of individuals.

Overall, the experimental results indicate that the proposed gait-phase-aware admittance controller delivered well-timed assistance throughout the gait cycle and produced a significant reduction in muscle activity across all five recorded lower-limb muscles, even with a maximum support torque limited to ±2 Nm.

## 4. Discussion

The experimental results showed that the proposed controller produced well-timed assistance synchronized with natural hip power generation events, consistently attenuating muscle activation across all five recorded lower-limb muscles. The reduction was significant for each muscle after correcting for multiple comparisons. Crucially, these outcomes were achieved without subject-specific tuning or external optimization, only by using the moderate support torque (±2 Nm). These observations suggest that gait-phase-aware adaptation, rather than a high assistance magnitude, serves as the primary catalyst for the observed muscular unloading.

Two specific control strategies were likely responsible for the observed reduction in muscle activation. The first is the synchronization of assistance with natural hip power generation events. The mechanical power profile in Figure 5d shows that the assistive power peaks are aligned with late-stance push-off and late-swing limb deceleration, which are the two phases in which the hip joint naturally produces positive work during healthy walking. Because assistive torque is delivered during these power generation events, it directly reduces the muscular effort required by the user. The second is a seven-subphase scheduling with smooth transitions. As noted in Section 2.4, a single fixed parameter set cannot match different mechanical demands across the gait cycle. By scheduling the parameters across the seven subphases, the controller adapted to each part of the cycle, and the two-stage rule prevented torque discontinuities at the boundaries. Together, these two strategies caused the assistance to follow the natural biomechanical pattern of walking rather than imposing a fixed torque profile.

The mean EMG WL reductions of approximately 19% to 24% observed in the proximal muscles (RF, BF, and VM) during the stance phase were highly consistent with the assistance timing, which peaked near the heel strike and during the late stance phase. During these periods, the hip joint naturally generates positive mechanical power; thus, the assistive torque supplied by HealBot-H effectively reduces the muscular effort required for power generation. The SL muscle serves as the primary plantar flexor during push-off. Its stance phase reduction is significant because the muscle is heavily loaded during this interval, and its baseline magnitude is inherently large. Conversely, during the swing phase, the SL is physiologically inhibited to facilitate ankle dorsiflexion [51]; therefore, its swing-phase attenuation has limited functional interpretation. Furthermore, the TA exhibits a characteristic biphasic activation pattern, with peaks near the heel strike for impact absorption and during swing for foot clearance [39]. Both activation peaks were distinctly suppressed under HealBot-H conditions. Notably, the finding that distal muscles such as the SL and TA also responded to hip-level assistance suggests that the proposed controller not only offloads the hip joint but also modifies the overall gait mechanics. This system-level cooperative response aligns with the expected behavior of the adaptive controller, which follows the natural gait pattern rather than imposing a predefined torque profile.

Recent studies reported EMG reduction with various hip exoskeleton designs. Cao et al. [52] reported reductions of 15.3% (lateral SL), 11.1% (medial SL), and 3.7% (gluteus maximus) in a multi-joint active-passive exoskeleton, with a metabolic reduction of 10.4%. Xu et al. [53] reported a 16.0% reduction in RF activity when using a portable hip exoskeleton with human-in-the-loop optimization. Kim et al. [54] reported a 7.2% metabolic reduction with a 2.31-kg soft hip-flexion exosuit. Although a direct cross-study comparison is precluded by distinct robotic architectures, assistance strategies, and experimental protocols, these references provide a context in which the EMG-level results of HealBot-H stand in the field. The findings of the present study suggest that a well-timed gait-phase-aware admittance controller integrated with a lightweight wearable platform can yield meaningful muscle activation reductions even with a moderate assistance torque and without any subject-specific tuning.

Beyond muscular unloading efficacy, this study demonstrates the real-time viability of implementing phase-aware adaptive assistance on a lightweight wearable platform. The seven-subphase scheduling framework provides a highly structured action space, whereas the dual gait-phase estimation modules offer flexibility of sensor configurations and a fallback path for stability. Furthermore, the successful real-time deployment of the deep learning estimator on the embedded hardware confirms that such computationally demanding control is feasible for a 2-kg robot. The framework also generalizes across users within the tested population. The same parameter table was applied to every participant, and the controller generates assistance from the estimated interaction torque rather than from a prescribed trajectory. Therefore, the assistive motion follows the walking pattern of the individual user. Taken together, these elements position HealBot-H as an adaptive assistance platform rather than as a fixed-torque device.

Nevertheless, this study had several limitations. First, although the built-in actuator could output a higher torque, the assistive torque was conservatively capped at a moderate level to ensure user safety and comfort. Therefore, the presented results indicate only a single operational point within the achievable assistance range. Second, the physiological evaluation relied only on surface EMG. The metabolic cost was not measured, and EMG was used as the primary proxy for the effectiveness of assistance. Future studies should combine EMG with metabolic measurements and subjective effort ratings to provide a more complete evaluation. The controller parameters were also selected by trial and error rather than by formal optimization. Third, the evaluation was limited to level overground walking under laboratory conditions. The robustness of the system was not investigated for varying walking speeds, long-term operation, or different environmental conditions. Each participant completed a single session, so comfort over extended wear, cumulative fatigue, and user adaptation were not assessed. Fourth, the gait phase estimator was trained on healthy walking data. Pathological gait patterns such as reduced range of motion, marked asymmetry, or a very slow cadence may reduce the accuracy of the phase estimate and therefore the timing of the assistance. Retraining or fine-tuning the estimator with data from the target population would be required before clinical use.

For future work, the platform developed in this study opens several promising directions. The seven-subphase parameter set forms a structured action space that aligns naturally with reinforcement learning (RL). In this approach, a hand-tuned parameter table can be replaced with an agent that assists each user through real-time biomechanical and physiological feedback. The deep learning gait phase estimator already supports stair ascent and descent at the model level; therefore, additional terrain conditions can be tested on the same hardware without adding new sensors. The modular structure allows the robot to be worn in a unilateral configuration, making HealBot-H suitable for hemiplegic patients and other cases in which assistance is required only on the affected side. Together, these directions extend the platform from level-walking assistance to personalized, multiterrain, and clinically targeted use. In addition, a systematic ablation quantifying the individual contribution of the subphase resolution, the blending stages, and the gait phase estimator is planned to further isolate the effect of each component.

## 5. Conclusions

This study presented the design, implementation, and experimental validation of a gait-phase-aware admittance control framework for HealBot-H, a 2-kg lightweight modular hip assistance robot. The main goal was to integrate a deployable wearable platform with a deep learning-driven adaptive controller in a single system. The Bi-LSTM gait phase estimator was deployed in real time on the RPi 5 and integrated with an admittance law, whose virtual inertia, damping, and stiffness were scheduled across seven subphases of the gait cycle. A two-stage update rule combining linear interpolation with exponential smoothing kept the parameter updates continuous at the subphase boundaries. The system was validated in ten healthy adults during overground walking. The assistive torque aligned with the natural hip power-generation events, and surface EMG was reduced by approximately 19% to 24% across five lower-limb muscles, without subject-specific tuning and with a moderate support torque of ±2 Nm. These results demonstrate that gait-phase-aware admittance control on a lightweight wearable platform can provide effective and well-timed walking assistance. The proposed framework also establishes a practical foundation for future personalized assistance using RL, multiterrain operation, and asymmetric gait rehabilitation.

## Figures and Tables

**Table 1:** HealBot-H specifications.

Specification	Value
Total weight (including battery)	2 kg
Continuous joint torque	10.3 Nm
Peak joint torque	43.8 Nm
Active DOF	2 (bilateral hip flexion/extension)
Passive DOF	2 (bilateral hip abduction/adduction)
Gearbox	Harmonic drive, 1:100
Joint angle sensor	Incremental encoder
Motor drivers	Maxon EPOS4 Compact 50/5 CAN
Modular connection	Magnetic quick release

**Table 2:** Stance-phase mean EMG WL values of various muscles for the *No-HealBot-H* and *HealBot-H* conditions.

Muscle	No-HealBot-H Mean ± SD (μV)	HealBot-H Mean ± SD (μV)	Difference (%)
RF	1.94±0.57	1.54±0.42	−19.59 **
BF	2.67±1.05	2.11±0.73	−19.75 **
VM	2.52±1.11	1.93±0.87	−23.41 **
SL	9.22±3.39	7.23±3.05	−21.94 **
TA	7.53±4.18	5.63±2.61	−23.52 **

Statistical significance was assessed using the Wilcoxon signed-rank test with Benjamini–Hochberg FDR correction (** $p_{FDR}<0.01$).

## Data Availability

Raw data supporting the findings of this study are available upon request from the corresponding authors.

## Supplementary Materials

The following supporting information can be downloaded at: https://www.mdpi.com/article/10.3390/s26165008/s1, Figure S1: Response of the proposed phase-adaptive admittance controller during a representative walk-turn-walk trial; Figure S2: Response of the fixed-parameter admittance controller during the same walk–turn–walk protocol.

## Abbreviations

The following abbreviations are used in this manuscript:

Bi-LSTM	Bidirectional Long Short-Term Memory
BF	Biceps Femoris
CAN	Controller Area Network
DOF	Degree(s) of Freedom
EMG	Electromyography
FDR	False Discovery Rate
HRI	Human–Robot Interaction
IMU	Inertial Measurement Unit
ONNX	Open Neural Network Exchange
RF	Rectus Femoris
SL	Soleus
TA	Tibialis Anterior
VM	Vastus Medialis
WL	Waveform-Length
RPi 5	Raspberry Pi 5
IRB	Institutional Review Board
RL	Reinforcement Learning
