# Supplementary material for: Embedded Adaptive Admittance Control for a Modular Hip Exoskeleton Using Deep Learning-Based Gait-Phase Estimation
Source: Sensors (Basel). 2026 Aug 7;26(16):5008. doi: 10.3390/s26165008 (PMC13517246; doi:10.3390/s26165008)
Supplement: Supplementary file 1 [file sensors-26-05008-s001.zip › sensors-4441553-supplementary.pdf]

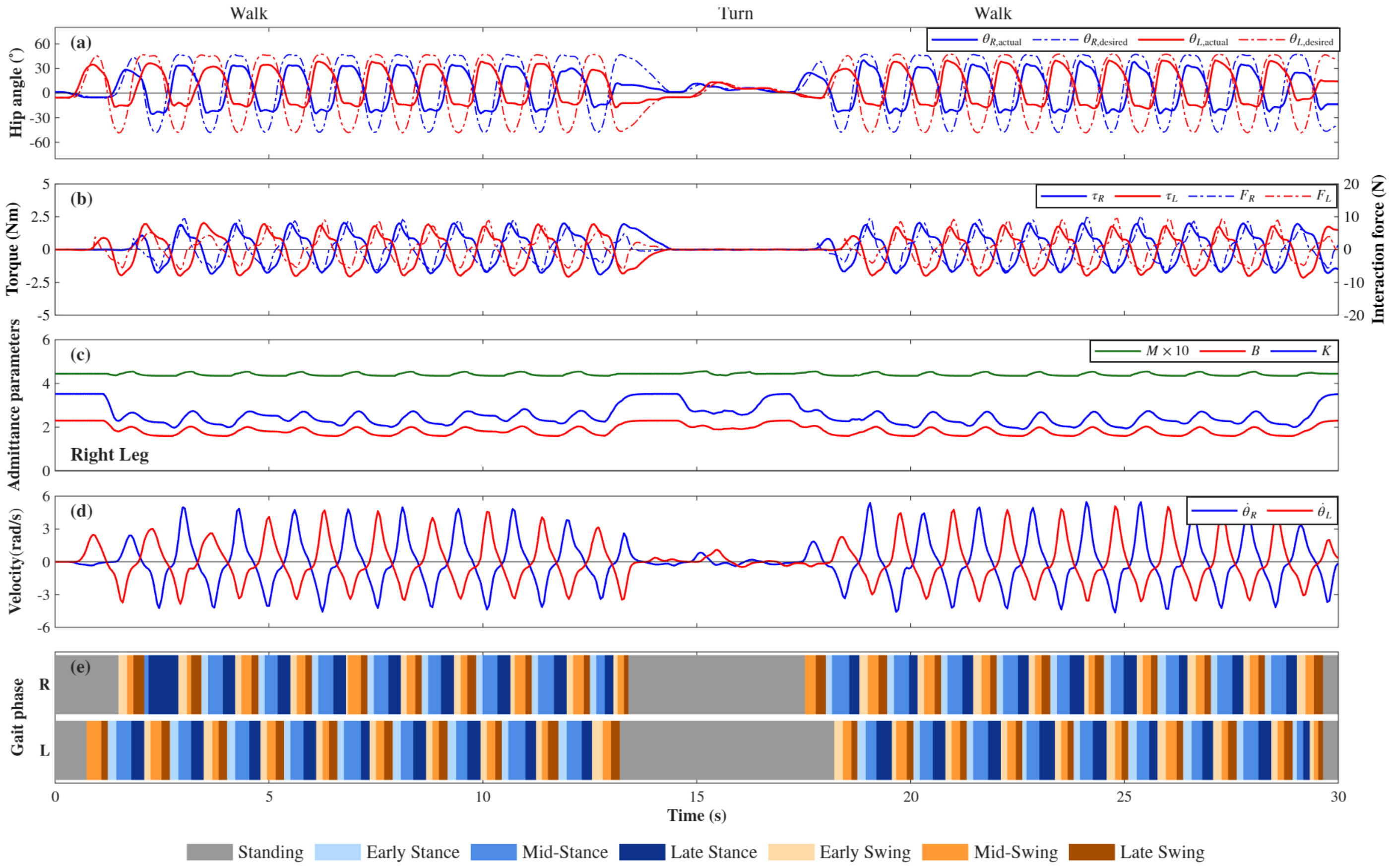

Figure S1. Response of the proposed phase-adaptive admittance controller during a representative walk-turn-walk trial. **(a)** Bilateral hip joint angles, where dashed lines show the desired trajectories generated by the admittance dynamics (θ_d) and solid lines show the measured joint angles (θ_{actual}). **(b)** Motor assistive torque (left axis) and estimated interaction force (F_R, F_L, right axis). **(c)** Admittance parameters for the right leg, where M is plotted at ten times its actual value for visual clarity. **(d)** Hip joint angular velocity. **(e)** Gait phase identified by the deep learning classifier, color-coded for the seven subphases.

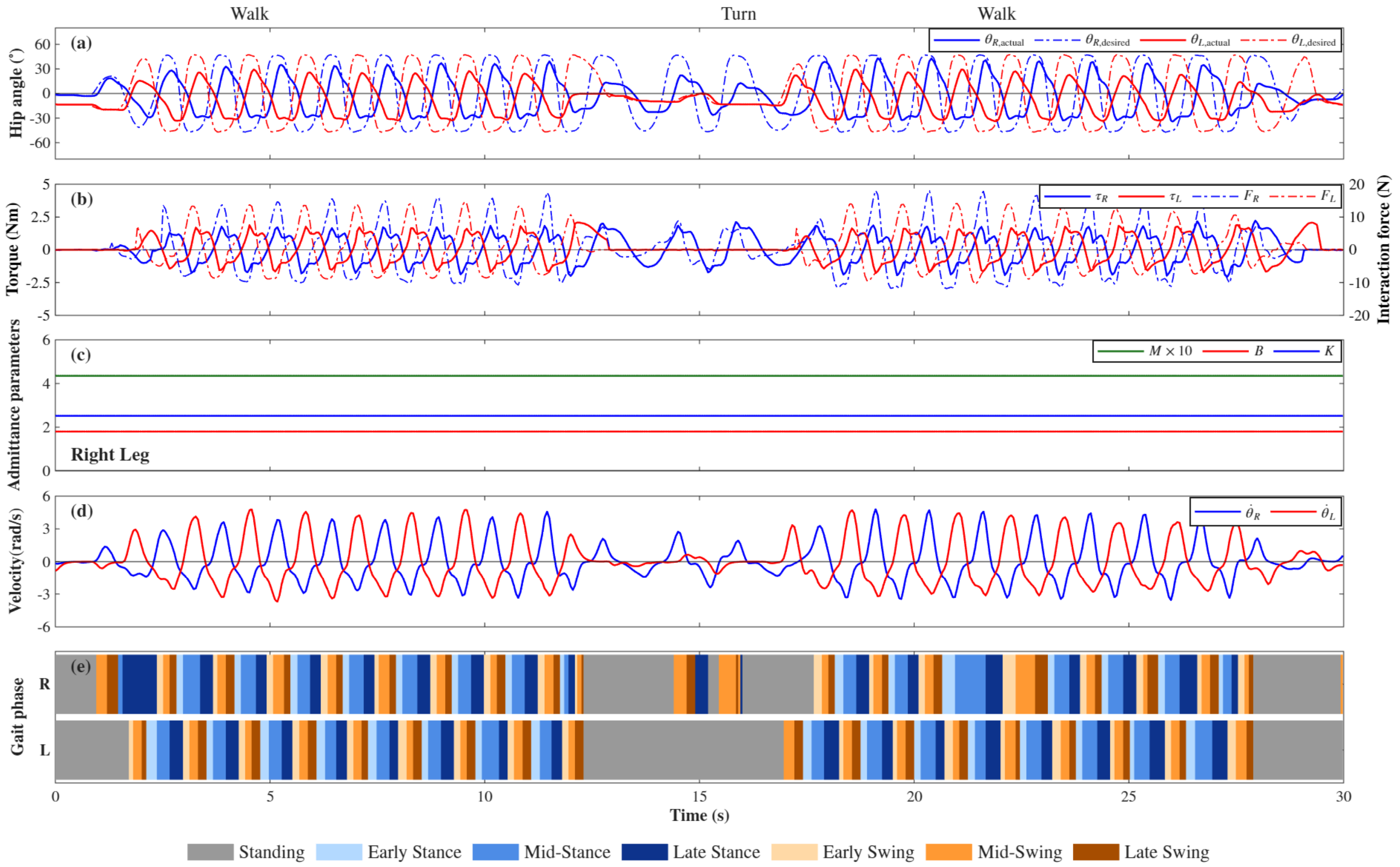

Figure S2. Response of the fixed-parameter admittance controller during the same walk-turn-walk protocol. The admittance parameters M, B, and K were held constant at the mid-stance values of the schedule used in Figure S1. **(a)** Bilateral hip joint angles. **(b)** Motor assistive torque (left axis) and estimated interaction force (right axis). **(c)** Admittance parameters for the right leg, shown as constant values. **(d)** Hip joint angular velocity. **(e)** Gait phase identified by the deep learning classifier.

A comparison between the proposed phase-adaptive admittance controller and a fixed-parameter admittance controller was performed during the same overground walk-turn-walk protocol. Both conditions used identical hardware, the same gait phase classifier, the same interaction torque observer, and the same inner torque control loop. The only difference between the two conditions was the source of the admittance parameters. Figure S1 shows the response of the proposed controller, in which M, B, and K were modulated across the seven subphases. Figure S2 shows the response of the fixed-parameter controller, in which M, B, and K were held constant at the mid-stance values of the same schedule. The mid-stance admittance parameter set was selected as the baseline condition for two reasons. The mid-stance values lie within the interior of the range spanned by the full schedule rather than at its extremes, so the fixed baseline represents a compromise setting rather than a setting biased toward failure. Mid-stance is also a weight-bearing subphase, which makes its parameters the conservative choice when a single setting must serve the entire gait cycle.

The objective of the admittance layer is to reduce the physical interaction between the user and the device, so the interaction force is the primary quantity of comparison. It is plotted against the right-hand axis of panel (b) in both figures. The peak interaction force reached approximately 10 N under the proposed controller in Figure S1(b) and approximately 18 N under the fixed-parameter controller in Figure S2(b) over the same protocol. The increase was not uniform across the gait cycle. It was concentrated at the transitions between the stance and swing subphases, where the mismatch between the fixed

admittance and the mechanical requirement of the subphase is largest. Stance requires higher stiffness and damping to support body weight, whereas swing requires low impedance so that the limb can move freely. A single fixed parameter set cannot satisfy both requirements, and the resulting residual force appears at the interface between the device and the user.

The turn-around interval revealed a second difference between the two conditions. Turning is not a cyclic gait event, and the controller should suppress assistance when no consistent gait pattern is present. In Figure S1(e), the classifier identified the turnaround interval as standing. The controller applied the standing parameter set, and the commanded torque in Figure S1(b) attenuated to near zero for the duration of the turn, so the device did not act against a maneuver that requires free, voluntary movement of the hip. In Figure S2(e), the classification during the same interval fluctuated between subphases, and no sustained standing state was produced during the turn. Figure S2(b) shows that the controller continued to deliver assistive torque throughout the interval. The commanded torque followed a walking pattern that no longer corresponded to the user's actual movement, and the interaction force remained elevated for the duration of the interval. Because the gait phase classifier operates on inertial signals that depend on the resulting limb motion, the controller lies within the input path of the classifier. The continued delivery of mistimed assistance may therefore have reinforced the initial misclassification, although this coupling was not tested directly in the present trial. Assistance delivered against a voluntary movement is expected to increase user effort, which suggests that fixed parameters degrade the quality of the interaction during non-cyclic maneuvers.
